# An ERP study on verb bias and thematic role assignment in standard Indonesian

**DOI:** 10.1038/s41598-025-96240-y

**Published:** 2025-04-07

**Authors:** Bernard A. J. Jap, Yu-Yin Hsu

**Affiliations:** 1 School of Arts and Social Sciences, Hong Kong Metropolitan University, Kowloon, Hong Kong; 2 Department of Chinese and Bilingual Studies, The Hong Kong Polytechnic University, Kowloon, Hong Kong

**Keywords:** Verb bias, Passive construction, Sentence comprehension, Standard Indonesian, Event-related potential (ERP), Human behaviour, Language

## Abstract

This study investigates the effects of verb bias on sentence comprehension in Standard Indonesian (SI), a language where the passive construction is frequent and salient. We examined the hypothesis that lexical biases influence processing efforts. To disentangle effects of verb bias from structural effects, we held structure constant by using only passive sentences in our event-related potential (ERPs) experiment. Using an SI corpus, we identified verbs more frequently used in passive sentences (“passive-bias verbs”) and those more frequently used in active ones (“active-bias verbs”) to form passive sentences. Cluster-based permutation tests revealed significant differences between conditions. In the postverbal region (adverb), passive sentences with active-bias verbs elicited a broadly distributed negative shift that corresponded to the N280 distribution, a component associated with grammatical processing complexity. This suggests that active-bias verbs in passive contexts impose greater processing demands than their passive-bias counterparts, reflecting the integration of verb-specific preferences with sentence structure. These findings highlight how verb bias influences sentence comprehension and provide insights into cross-linguistic differences. In languages with predominantly active-bias verbs, using them in passive constructions may amplify processing costs. This study shows the importance of lexical biases and structural preferences in shaping sentence processing, contributing to a broader understanding of the interaction between lexical and syntactic factors in language comprehension.

**Supplementary Information:**

The online version contains supplementary material available at 10.1038/s41598-025-96240-y.

## Introduction

Many languages convey who does what to whom through information provided in verb phrases. For example, the English sentences in examples (1) and (2) show that *the man* is affected by the action *push*. However, in example (3), the word “raced” is often initially read as a main verb, but it must be re-analyzed as a passive participle upon encountering “fell.”^1^ This type of sentence is known as a garden path sentence because it initially leads the reader down an incorrect parsing path, requiring reanalysis. The initial misinterpretation arises because the initial part of sentence structure is ambiguous: it can be interpreted either as a simple sentence with “raced” as the main verb, or as a complex sentence with a reduced relative clause (“raced past the barn”) modifying the subject “horse.”

1. The man was pushed by the woman.
2. The woman pushes the man.
3. The horse raced past the barn fell.

Previous studies of sentence processing have often compared active and passive sentences without adequately controlling for verb bias, despite evidence that both syntactic frequency and verb bias affect sentence comprehension^2^. In English, for instance, active sentences are overwhelmingly more frequent than passive ones, and the agent-action-patient order is therefore that language’s dominant pattern for expressing thematic roles; i.e., most English verbs bias readers towards an initial active reading. This is common in many syntactic structures, as individual verbs exhibit biases based on whether they are more often used with one structure or the other.

This contextual conditioning of word probabilities has prompted the exploration of other types of *conditional probabilities*, i.e., estimates of the likelihood of a given outcome based on a given circumstance. When conditioned on specific verbs, estimated probabilities are often referred to as “verb bias”. The importance of verb biases quickly became evident in research of sentence comprehension, particularly in sentences with structural ambiguity. A series of studies has established that verb biases^6–9^ and other lexically conditioned probabilities significantly impact sentence comprehension. However, previous studies often conflated verb bias effects with those of syntactic structure. For example, “constructional frequency” refers to processing differences linked to the relative frequency of constructions, such as active versus passive^10,11^ or subject versus object cleft^14,15^. These effects are often treated as inherent to the constructions themselves. Notable, exceptions have been observed in Austronesian languages including Standard Indonesian^12^ and Tagalog^13^.

The tendency to compare syntactic constructions without isolating verb bias has made it difficult to disentangle the contributions of these factors. For example, difficulties in processing garden-path sentences^3,4^ are not solely due to the relative frequency of intransitive vs. transitive constructions in general, and it is unrelated to the relative frequency of intransitives vs. reduced relative clauses, as demonstrated by the ease of processing sentences like “The sign posted on the fence says to keep out.” In such sentences, like (3), the verb “race” is far more often used as intransitive than transitive^5^, making it intransitively biased and thus biased for the main clause reading.

While the greater frequency and processing ease of active transitive sentences compared to passive ones is well-documented, these differences may arise not only from constructional frequency effects but also from lexical factors such as verb bias. For instance, the verb “elected” is more commonly used in passive constructions, whereas “kissed” tends to be associated with active ones. Lalami^16^ found that neurotypical speakers’ accuracy at comprehending passive constructions varied based on the relative frequency of passive and active verbs. Furthermore, sentences with passive bias verb were easier to process in passive constructions than those with active ones^10,17^. For example, a sentence like “The candidate elected for governor was very pleased” is easier to comprehend than one like “The candidate elected to change the topic.” This pattern of greater difficulty for passives compared to actives is most obvious when highly transitive, active-biased verbs are use in the experimental materials.

Therefore, the current study addresses this research gap by isolating the effect of verb bias on sentence comprehension. By keeping syntactic structure constant (using only passives) and manipulating verb preference, we avoid the structural frequency effects that have dominated previous research. Using an SI corpus^18^, we focus only on effects of verb bias, offering a novel contribution by disentangling the processing effects of structural frequency and verb bias.

## ERP studies on sentence processing

ERP studies on sentence processing have explored neural differences in processing various word orders and syntactic complexities. The N280, an early negative-going component peaking around 280ms post-stimulus with an anterior distribution^42^, has been associated with grammatical processing complexity rather than simple frequency or categorial effects^42^. Brunellière et al.^42^ demonstrated that N280 amplitude increases with syntactic complexity, even when controlling for word length, frequency, and lexical category. Additionally, the absence of N280 in patients with Broca’s aphasia, who struggle with grammatical processing^70^, further supports its role in syntactic processing.

Consistent with the behavioral results, previous ERP studies find that object-first structures are more challenging to comprehend than subject-first structures^19^. For example, Matzke et al.^20^ observed a Left Anterior Negativity (LAN) component for object-first structures, and a P600 in the disambiguation region of such sentences in German, and this LAN effect observed for object-first sentences was attributed to increased working memory demands. The LAN, a negative-going component occurring 300-500ms post-stimulus with a left-anterior distribution, has been linked to morphosyntactic violations^43^ and syntactically complex in well-formed sentences, particularly those with non-canonical word orders that increase working memory load^20,21^. The P600, a positive-going wave appearing 500-800ms post-stimulus with a centro-parietal distribution, reflects syntactic violations^44^ as well as structural reanalysis and integration processes of well-formed sentences^45^. Schlesewsky et al.^21^ found LAN only in object-first non-pronominal first noun phrases (NP1) in German, suggesting that it reflects both working memory demands and syntactic mismatches in processing non-canonical word orders.

Other studies have identified similar patterns across languages. Meltzer and Braun^22^ found a P600-like positivity at the end of critical clauses for reversible versus irreversible sentences, followed by a left anterior negativity for sentences containing relative clauses (both subject relatives and object relatives) compared to simple active sentences. They did not find significant differences between the two types of relative clauses, although traditionally object relative clauses are considered more complex. Jackson et al.^23^ observed a positivity for passive over active structures in English. In Japanese, Wolff et al.^24^ reported an early negativity effect (scrambling negativity) and a late parietal negativity at the verb for object-initial structures. Similar effects were observed in Basque^25^, where object-first structures elicited a left-lateralized negativity and parietal positivity at the verb, indicating increased processing demands for scrambled sentences. The P600 reported for passive structures compared to actives^23^, suggesting that it indexes the additional processing demands of less-preferred syntactic structures.

Overall, the previous studies suggest that negative shifts occurred at NP1 for case-marked languages, although specific ERP components vary across studies, due to other factors such as verb biases, linguistic differences, and experimental design, making it difficult to pinpoint the neural correlate of processes involved in reading object-first structures, including thematic role processing.

## Passives in Standard Indonesian

Standard Indonesian (SI), used in Indonesia’s education system, government and other formal settings, belongs to the Western Malayo-Polynesian subdivision of the Austronesian language family. It has approximately 23 million native speakers and over 140 million second language (L2) speakers^26^. Originally, all Indonesians spoke what are now considered regional dialects as their first languages, and SI was only acquired as an L2; however, the number of native speakers of SI continue to grow^27^.

SI is a zero-marking language^28^. This means that it does not use morphological markers on the core arguments of a predicate^29^. Also, SI’s transitive verbs are typically inflected only for voice (active or passive). Aside from some reduplicated verb constructions that signify iteration^30^, SI does not use verb inflection for tense, aspect or agreement. For example, the verb *memasak* in example (4b) provides a lexical entry and indicates voice (active) as well as transitivity.

A base sentence in SI has two obligatory components: the subject and the predicate. The subject of a sentence is generally a noun phrase, and is also often what is being discussed (i.e., the topic; as such, it is often produced in the form of a pronoun), though nominal clauses can also appear in the subject position. The predicate can be either non-verbal (4a) or verbal (4b).

(4) a. Andi di rumah.

Andi at home

         "Andi is at home."

b. Andi **me**masak nasi.

   Andi **ACT-**cook nasi

   "Andi is cooking rice."

SI’s lack of morphological marking requires it to have a relatively rigid word order. However, in certain constructions such as *wh*-questions^31^ and predicate nominalization^32^, the ordering of constituents can be flexible. The base word order in an SI clause is subject-verb-object (SVO). Like English, SI also has simple active sentences (5a) and simple passive sentences (5b).

(5) a. Simple active (agent-theme).

Perempuan itu      **me**manggil laki-laki itu.

girl the  **ACT**-call boy the

"The girl is calling the boy."

b. Simple passive (theme-agent).

*Laki-laki itu* ***di****panggil  (oleh) perempuan itu*.

boy the **PAS**-call  (by)  girl the

"The boy is called by the girl."

Example (5a) is a simple sentence with an active-voice marker (*meN-*, reduced to *me-* due to assimilation) on the verb. The sentence in (5b), like the materials used for testing in our study, can be semantically ambiguous (due to the reversable interpretations of two animate NPs); it is expressed by the *di-* prefix, as on the verb in (5b), in which “the boy” is the theme. Partly similar to the optional *by*-phrase in English passives, both spoken and written SI allow the preposition *oleh* (“by”) to be omitted when nothing intervenes between the passivized verb and the agent phrase. When the agentive preposition is omitted, the noun phrase-verb-noun phrase (NP–V–NP) structure of the passive appears to be the mirror image of the active structure.

The passive construction appears to play a more critical role in SI than in many languages. It is often acquired as early as two year old^33^, compared to the English passive, typically acquired around ages four or five^33^. In part, this early acquisition may be because of the higher input frequency of passive structures in SI, estimated at 28–35%, as compared to 4–5% in English passive. As they age, Indonesian adults also use SI passives more frequently in both spoken and written contexts^33^. Another notable contrast with English has been highlighted by a corpus study^15^. English passives appear four to five times more often in written corpora than in spoken corpora, indicating that the English passive is primarily a written structure. In written SI, however, 30–40% of verbs carry the passive *di-* prefix^34^, compared to just 9% of written English verbs with passive morphology^35^. A high frequency of passives is also found in Classical Malay, from which SI is derived, as well as in other Malay languages^36^. While comparable spoken corpora for SI may not be available, evidence from early acquisition and the frequency of adult spontaneous speech data^33^ suggests that the passives in SI are also common in spoken contexts. The saliency of the passive in SI can be attributed to its unambiguous voice morphology^12^, which the *meN-* prefix indicates active and the *di*-prefix marks passive. Additionally, the active prefix *meN-* contains a schwa and is often reduced in speech through stem-initial assimilation (e.g., *menyapu* ‘to sweep’ -> *nyapu* ‘to sweep’), making the passive prefix *di-* more prominent in comparison. This clear distinction provides more straightforward cues for language learners and comprehenders.

It should be noted that the passive voice in Indonesian has a functional use: it enhances politeness. Randriamasimanana^37^ recorded and analyzed the usage of passive verbs in letters written by adult L1 SI speakers in the late 1970s. One of the sampled individuals wrote three letters: one to their eldest son, one to a younger son, and one to a civil servant. The letter to the civil servant contained a much higher proportion of passive verbs (57.1%, 32/56) compared to the letters to the sons (eldest son: 29.5%, 18/61; younger son, 16.3%, 8/49).

Beyond politeness, however, frequent use of passives in SI is also motivated by specific discourse functions that are distinct from those of active verb forms. Based on discourse analysis, Kaswanti-Purwo^38^ identified the key functions of *di-*verbs, such as foregrounding, describing punctual or factual events, and introducing actions that come in sequences. In contrast, *meN-*verbs were found to be associated with backgrounding or initiating discourses, describing habitual or nonfactual events, providing parenthetical information, and breaking or closing narrative flows. Verhaar^39^ also noted some contexts with which *di-* passives are more “compatible,” for instance, when the verb form is not reduplicated or when the sentence does not specify duration.

Building on previous research, we examine passive sentences with either active-bias and passive-bias verbs in an Austronesian language – Standard Indonesian, using typical, plausible (both NPs are animate), violation-free sentences. To the best of our knowledge, no prior ERP study has investigated verb bias in Austronesian languages, despite their high frequency of passive-bias verbs compared European languages. This is important both theoretically and practically. Theoretically, past studies comparing active and passive sentences often used active-bias verbs in both cases, overlooking evidence that verb bias plays a crucial role in sentence processing^7,17^. Investigating passive-bias verbs in Austronesian languages could enhance understanding of how verb bias affects language comprehension. Practically, Standard Indonesian remains understudied in neuro-psycholinguistics despite its large speaker base.

## The present study

This study investigates the processing of simple passive sentences with contrasting verb biases during sentence comprehension. Particularly, we examine the verb and the postverbal area, including the word immediately after the verb and (*by*-)NP2, to capture sustained spill-over effects. Prior studies have reported extended ERP effects beyond the disambiguating region that assigns thematic role. For instance, in Japanese, where nominative and accusative cases are marked on the noun phrases, varying latencies of negative ERPs on the verb of object-first structures (i.e., the post-disambiguation area, as case marking already reveals the thematic roles of the noun phrases) have been reported: 200–600 ms^40^, 300–900 ms^41^, and 600–1000 ms^24^. These significant differences led Wolff et al.^24^ to suggest that object-first sentences impose higher processing costs, as reflected in behavioral measures like increased error rates and reaction times.

We hypothesize that passive sentences formed with *active-bias verbs* will incur greater processing costs than those formed with passive-bias verbs due to the mismatch between the verb’s frequent active usage and its passive morphology. Specifically, we anticipate the following:

1. ERP amplitude: (a) Passive sentences with active-bias verbs will elicit larger negative shifts (e.g., LAN or N280) than those with passive-bias verbs, reflecting increased processing demands for using a verb in its non-preferred syntactic environment, similar to the LAN effects observed in non-canonical word orders^20,21^. (b) Additionally, if readers engage in structural reanalysis to resolve the conflict between the verb’s bias and the sentence structure, we anticipate a P600 effect similar to that observed by Jackson et al.^23^ for passive structures.
2. Timing: (a) The critical region introducing voice morphology and thematic roles (i.e., the verb) will elicit greater processing difficulty in passive sentences with active-bias verbs compared to those with passive-bias verbs. (b) Spill-over effects are expected on the immediately following word (i.e., an adverb in our stimuli) or the subsequent region (i.e., NP2), reflecting delayed integration or resolution of the structural mismatch.
3. Direction of effect: Passive sentences with active-bias verbs would be more difficult to parse, since the parser must resolve the mismatch between the unpreferred verb-bias and the passive morphology.

## Methods

### Participants

44 adult SI speakers with L1 or self-reported equivalent proficiency were recruited for this study. Data from 9 participants were excluded due to low trial counts after preprocessing and artifact rejection. The remaining 35 participants, who had at least 10 trials in both conditions in all three trigger points of the sentences, had a mean age of 39. All participants signed a written informed consent and were financially compensated HK$200 (about US$ 25.6). The study was approved by the Hong Kong Polytechnic University’s Institutional Review Board (ref no. HSEARS20211223003).

While some ERPs components, like the N400^46^, have been observed with large magnitudes (e.g., mean amplitudes over − 7 µV), they typically arise in a congruent/incongruent or violation paradigms, which generally elicit stronger effects than non-anomalous sentence processing^40,41^. Because the current study is an exploratory study and focuses on thematic role assignment and sentence processing, where findings across similar ERP experiments often diverge^19^ and no single ERP component seems to definitively index the processing of verbs in unpreferred but grammatical syntactic setting. To ensure sufficient statistical power in our analysis, we conservatively compared our target effect size to that of the lateralized readiness potential (LRPs), a typically smaller ERP component^47^ (around 1–4 µV) which has clear guidelines for statistical power^48^. Boudewyn et al.^48^ showed that 32 participants with 45 trials per condition are both (1) sufficient to achieve high internal reliability for the ERP (with Cronbach’s alpha of 0.7–0.9) and (2) sufficient to detect even relatively small ERP effects in within-subject designs. Their Monte Carlo between-condition simulations showed effects of 0.75 µV have over 90% probability while effects of 1 µV, 1.25 µV and 1.5µV all have peak (100%) probabilities of achieving *p* < .05. Effects larger than 1.5 µV will achieve peak probability with participant numbers as low as 20, whereas effects smaller than 0.75 µV (e.g., 0.5 µV and 0.25 µV) do experience improvement in the probability of detecting a difference with larger sample sizes (with the range between 12 and 32 participants) though it remains lower than 0.8 (80%). Based on these estimates, we believe that the present study’s 35 participants with 60 trials presented (after pre-processing, an average of 41.9 artifact-free trials per participant per condition) can detect subtle ERPs like those produced in non-violation paradigms^19^ and provides us with a reasonable statistical power to provided true differences between the conditions of at least 0.75 µV.

### Materials

The participants read stimuli comprising 120 passive sentences, of which 60 had passive-bias verbs and the other 60, active-bias verbs. Example stimuli are shown in Table 1 (see also S1 in the Supplementary Information).

**Table 1:** Stimuli examples from each condition.

Condition	NP1	Art	VP	Adjunct	*by*-NP2	PP
A-bias	Pria	itu	dilatih	kemarin	oleh staf	dari kantornya.
	Man	that/the	**PAS**train	yesterday	by staff	from his office.
	"(The/A) man was trained yesterday by the staff from his office."					
P-bias	Pria	itu	diberhentikan	kemarin	oleh wanita	karena perbuatannya.
	Man	that/the	**PAS**dismissed	yesterday	by woman	due to his behavior
	"(The/A) man was dismissed yesterday by (the/a) woman due to his behavior."					

NP = noun phrase; Art = article or demonstrative; VP = verb phrase; PP = prepositional phrase; A-bias = active bias; P-bias = passive bias; PAS = passive.

Additionally, there were 200 filler trials, comprising a mix of questions (e.g., *What did the adventurer notice yesterday?*; *n* = 100) and active sentences (e.g., *The woman met the man yesterday at the train station*; *n* = 80) to prevent habituation of the participants. All fillers were also well-formed sentences with no grammatical or semantic violations.

The materials were presented visually, word-by-word. Each trial began with a 750 ms blank screen, followed by the word *siap?* (“ready?”), which remained until the participant pressed a key. Each word in the sentence then appeared for 500 ms, seperated by 100 ms blank screens. Two pseudo-randomized sentence lists contain 10 blocks per list (each block containing 12 experimental sentences and 18 fillers) to minimize unintended syntactic priming across the stimuli^49^. Each participant only reads one list. Digital triggers marked three critical regions: the onset of the verb’s prefix, the onset of the adverb, and the onset of *by-*NP2.

All the experimental sentences used animate subjects and objects. To avoid prototypicality or plausibility bias, verb usage and NP order were reversed across trials. For example, if “the man” was NP1 in one sentence, it also served as NP2 in another sentence with the same verb. This design included 120 experimental sentences, using 60 unique verbs (30 active-bias and 30 passive-bias).

### Frequency

The main challenge of this study was to manipulate verb bias while controlling for the potential confounding effect of frequency. Specifically, we aimed to select active-bias and passive-bias verbs with a clear difference in their *active* voice frequencies, while keeping their *passive* voice frequencies as similar as possible. However, achieving perfect equivalence in passive frequencies between the two verb groups proved challenging. This highlights a broader issue that manipulating the relative syntactic frequency of a structure in a language may require intensive exposure to a certain structure or priming^50^. To address this, we selected verbs based on two criteria:

1. A higher token frequency in its active (passive) form compared to its passive (active) counterpart.
2. A higher frequency class in the active (passive) form compared to the passive (active) counterpart.

*Frequency class* is calculated by dividing the frequency of the most common word in the corpus by the frequency of a specific word, followed by the log base 2 of the result, rounded up^51^. Although we aimed for similar absolute passive frequencies for both verb types, this was not fully achievable in practice.

Previous ERP studies on passive sentence comprehension have often overlooked the fact that most verbs have much higher frequency in their active forms. Few previous studies also tried to address this issue; for example, Jackson et al.^23^ used past tense and progressive aspect (“-ing”) forms instead of simple present active structures. For our study, this type of adjustment was unnecessary, as we only used passive structure and manipulated only verb bias.

**Fig. 1:**
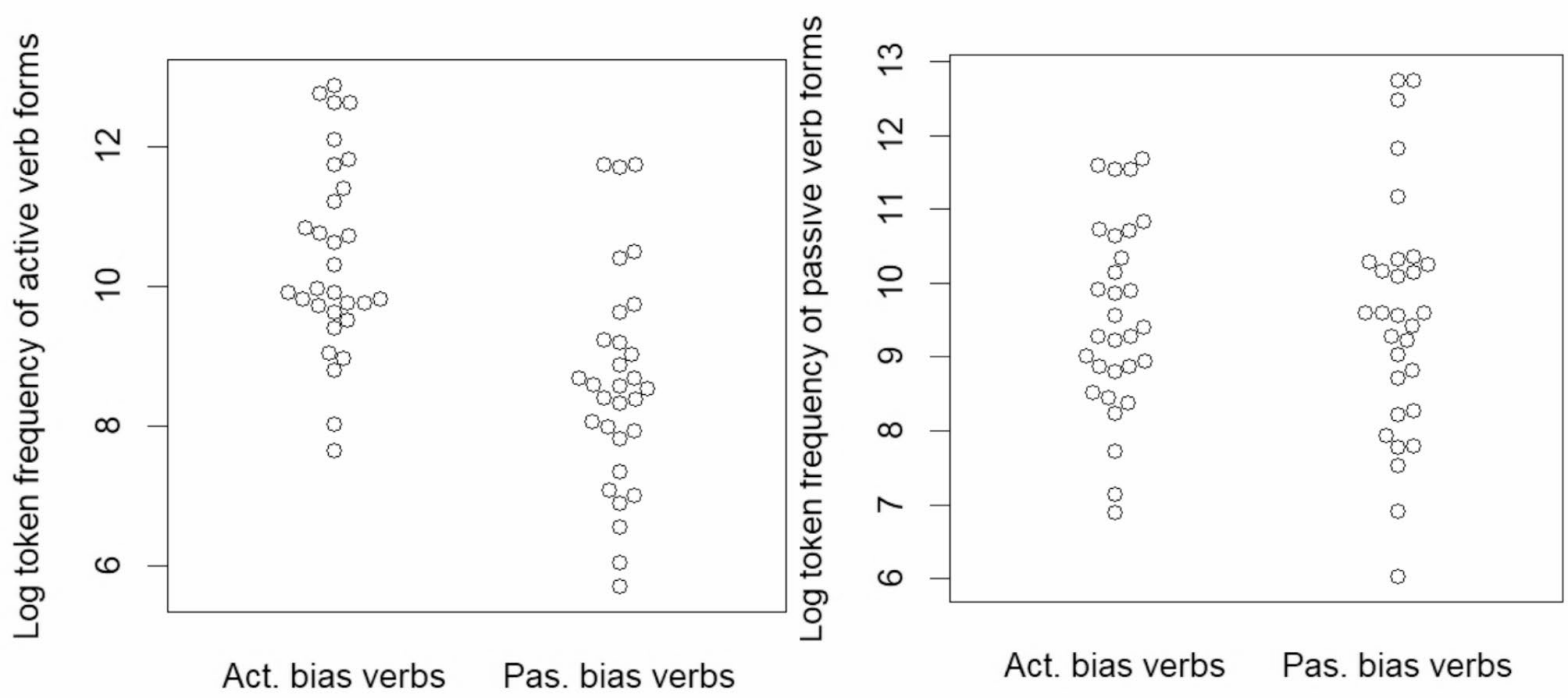
Beeswarm plots of log-transformed token frequencies for verbs selected for the current study. Each circle represents a single verb. (Left) Active verb forms: Active-bias verbs tend to have higher frequencies in the active voice. (Right) Passive verb forms: Passive-bias verbs tend to have higher frequencies in the passive voice.

We used the *Indonesian mixed corpus*, currently the largest online SI corpus in the Leipzig Corpora Collection^51^. Active-bias verbs had a mean passive frequency of 28,255, while passive-bias verbs had a mean passive frequency of 49,271. The frequency distribution and descriptive statistics are shown in Fig. 1 and Table 2, respectively. Log-transformed frequencies (Fig. 1) show distinct patterns between conditions: active-bias verbs are more frequent in active voice forms (left panel), while passive-bias verbs dominate in passive voice forms (right panel). Importantly, all passive-bias verb have higher passive-form frequencies than their active-form frequencies, and *vice versa* for active-bias verbs (Detailed frequency information is provided in S2 of the Supplementary Information).

**Table 2:** Descriptive statistics of passive verb form for active and passive-bias verbs.

	Active-bias	Passive-bias
Mean	28255.03	49271.13
Standard deviation	34864.2	94889.68
Range	117,999	342,184
Minimum	984	411
Maximum	118,983	342,595

## Procedure

Before the experiment, participants read an information sheet, completed a demographic questionnaire, and signed an informed consent form. Each participant was seated alone in front of a monitor running E-Prime 2.0 (Psychology Software Tools, Pittsburgh, PA). The experimenter gave oral instructions, which were also displayed on the screen. Prior to the start of the experiment, participants were instructed to minimize head movements and can blink naturally while avoiding prolonged eye closure during trials.

A fixation cross was shown between trials and sets of trials. After every three to eight trials (randomized), participants responded to a comprehension prompt. For experimental items and filler items, this was a *yes*/*no* question that probed thematic-role assignment (e.g., “Did the police shoot the robber?” or “Did the robber shoot the police?”). For some filler items that were questions, the participants were asked to judge whether the sentence shown on the screen was an appropriate response to the question. These prompts ensured participants stayed focused on reading the trials. To avoid fatigue, participants were allowed to take a break after each 30-trial block. The entire experiment, including preparation and breaks, lasted approimately 90 min.

## Electroencephalogram recording and preprocessing

EEG data were recorded using a 64-channel Quik-Cap Neo Net (Compumedics NeuroScan, Charlotte, NC) with Ag-AgCl following the 10–20 system. An integrated vertical electrooculogram (VEOG) above the wearer’s left eye were recorded. Two electrodes recorded signals from the left and right mastoids. To monitor horizontal and vertical eye movements, two further electrodes were fixed in the outer canthi of each eye, and one more was placed below the left eye. Electrode impedances were kept below 5 kΩ. Signals were amplified and digitized with a sampling rate of 1000 Hz using a SynAmps 2 (Compumedics NeuroScan, Charlotte, NC) with an analog bandpass filter set at 0.03–100 Hz. The recordings were conducted in a Faraday cage. A Cedrus Stimtracker provided an interface between the experiment’s presentation software and the acquired EEG data. Data were acquired using Curry 7 software that exported them as .cnt files for preprocessing in EEGLAB^52^ and analysis in FieldTrip^53^.

After re-referencing to the two mastoid electrodes and interpolation of bad channels, the EEG data were filtered using a 0.1 Hz high-pass filter. ERPs were calculated per participant, per electrode, and per condition in intervals lasting from 200 ms before onset to 1000 ms after onset for each time-locked trigger. These epochs were then de-meaned per channel in each epoch; i.e., the mean of the data from the entire epoch was subtracted from each data point, as this tends to result in better independent component analysis (ICA) decompositions than baseline correction based on pre-stimulus intervals^54^. ICA was performed using the *runica*() in EEGLAB^55^, and artifactual components (e.g., muscle or eye movement) were identified with the ICLabel algorithm^56,57^, and remove if their probability of being artifacts exceeded 0.9 (90%). This is a default conservative value recommended in optimized preprocessing pipelines^58^. A 200 ms pre-stimulus onset baseline correction was then applied.

Artifact detection used a moving-window peak-to-peak threshold function with a window size of 200 ms, a window step of 50ms, and a threshold of 75µV. Detected artifacts were removed, and the data were low-pass filtered at 40.0 Hz. Some studies (e.g., Wolff et al.^24^ and Friederici et al.^59^) are against baseline corrections in experiments of sentence processing because in mid-sentence time windows, the waves of each trial may not be identical prior to the onset of the critical word, and this has the potential to distort the baseline. Wolff et al.^24^ instead used narrower bandpass filters with higher low-end cutoffs (i.e., 0.3–20.0 Hz) to exclude slow drifts while still including language-related ERPs. However, Steinhauer^60^ criticized such alternative methods that first, the modified filter does not distinguish between artifacts (slow drifts) and real slow waves related to language processing; second, the filter converts sustained effects into apparent local effects such as early left-anterior negativity (ELAN); and finally, increased filtering does not directly address the problem of baseline distortion. We therefore adopted a 200 ms baseline in this study.

The signal-to-noise (SNR) ratio is an objective measure of EEG data quality and is a more impartial way of judging if a dataset has a sufficient level of evoked signal quality, compared to qualitative approaches or visual inspection^61^. While we did not use SNR ratio as an exclusion criterion, we report the SNR of participants included in the dataset using the bootstrap method by Parks et al.^61^. An SNR lower bound confidence-interval (*SNR*_*LB*_) value of 0dB or below indicates that the signal in the epoch/time window does not statistically exceed noise. The mean *SNR*_*LB*_ is 3.29 dB (SD = 1.14, Range = 1.72—6.11), exceeding the recommended threshold of 3.0 dB for high classification accuracy (> 90%).^61,62,63^ While SNR was not used as an exclusion criteria in our study, we used it to provide information on data quality^64^. Studies adopting SNR-based exclusion criteria often report significant participant loss (e.g., 16/72 participants retained in one study^65^), suggesting the threshold may be overly conservative. Nevertheless, SNR values depend on the experimental design and expected ERP magnitudes, with larger effects (e.g., violation paradigms) typically requiring higher SNR values^61^.

### Statistical analysis

Cluster-based permutation tests^66^ were used to analyze data across all scalp channels and the entire post-stimulus epoch (0-1000ms). This method allows testing for effects anywhere on the scalp and any time in the epoch, while still controlling the familywise false positive rate, avoiding the potential biases from selecting measurement windows and sensor sites^67^. This also allows us to detect ERP effects of varying latency, which is ideal for the exploratory nature of the present study. The analysis identifies clusters of spatiotemporally adjacent data points for which the differences between two conditions are significant, and then a permutation test evaluates the level of significance of the difference between those conditions, controlling for familywise error rate, as Maris and Oostenveld^66^ have demonstrated. In our analysis, data points were included in a cluster if they met a one-tailed threshold of *p* < .05 (cluster alpha), with at least two spatial neighbors. We ran two whole-epoch one-tailed tests: one for negative and one for positive polarity. The permutation test used 5000 iterations.

## Results

**Fig. 2:**
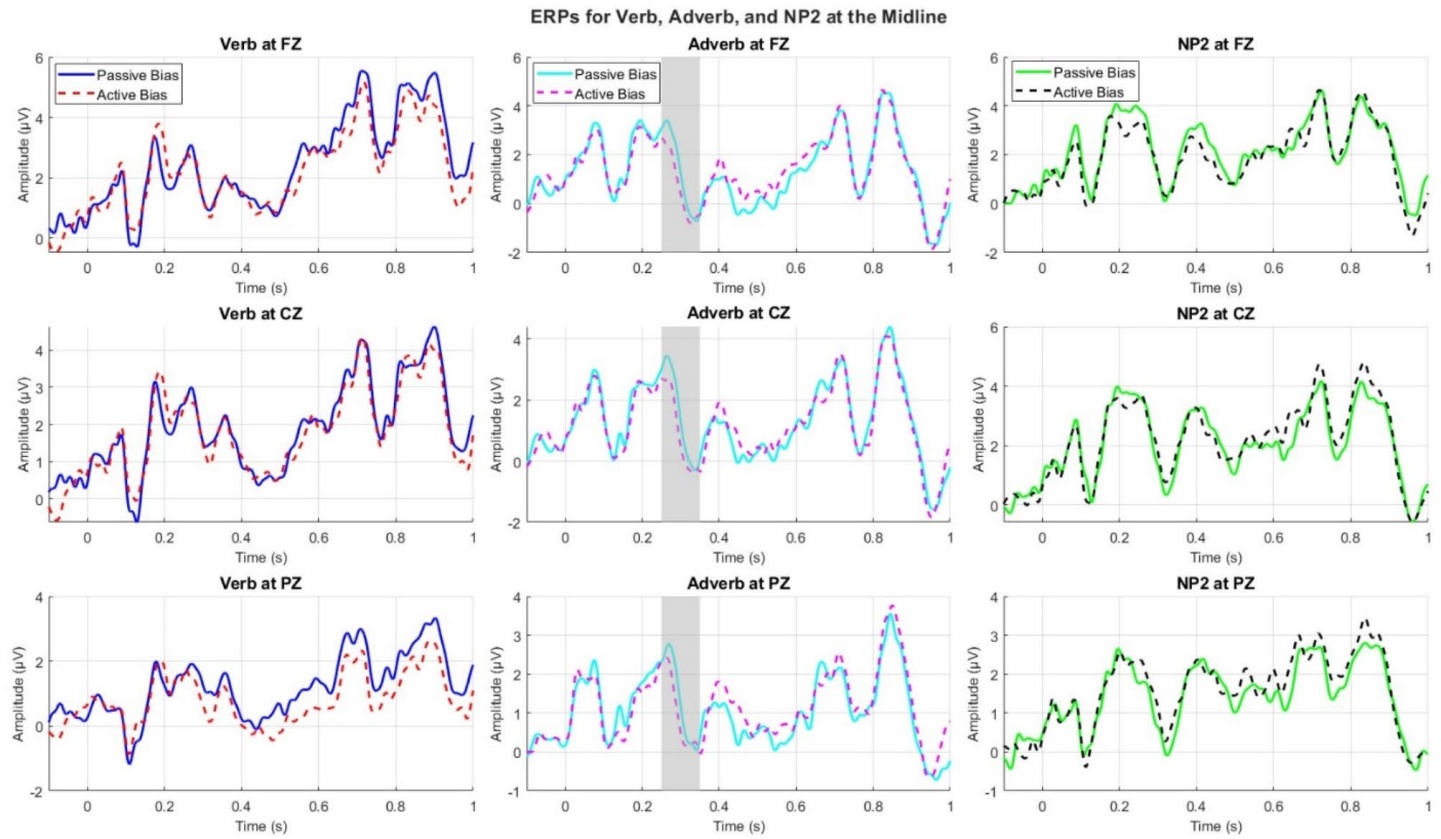
ERPs at the midline for all conditions.

**Fig. 3:**
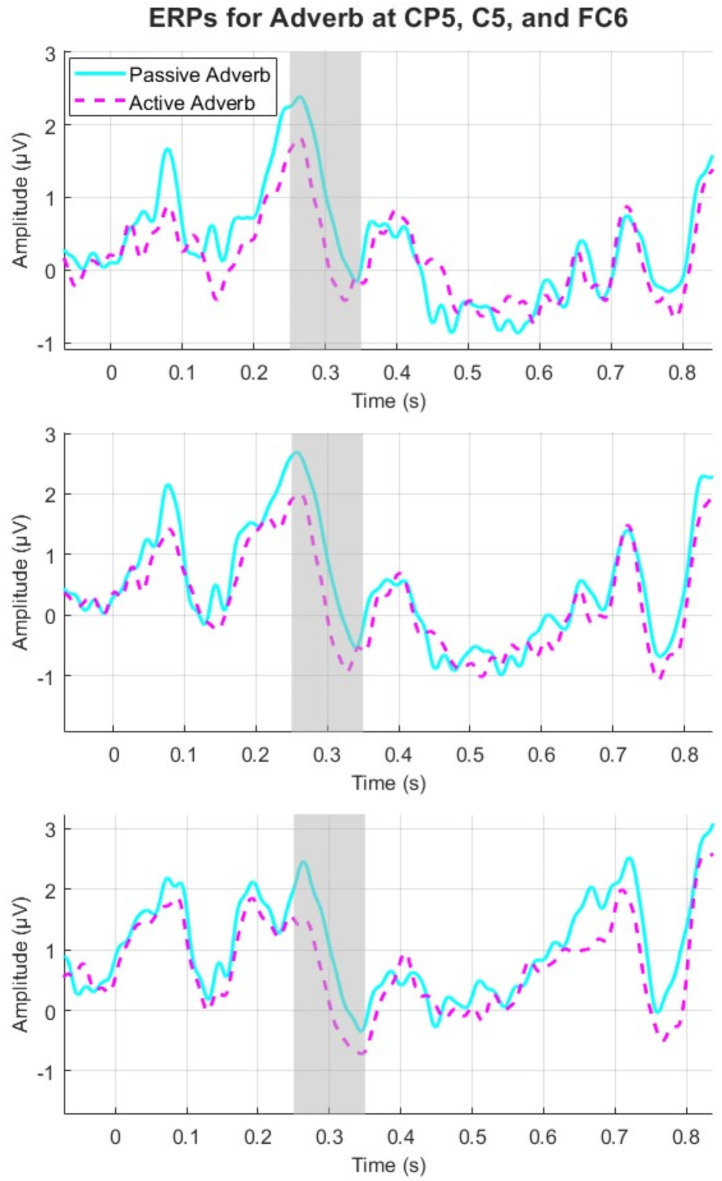
ERP at the adverb for channels CP5, C5, and FC6. These were chosen based on the cluster test results which show significant clusters at these electrodes.

**Fig. 4:**
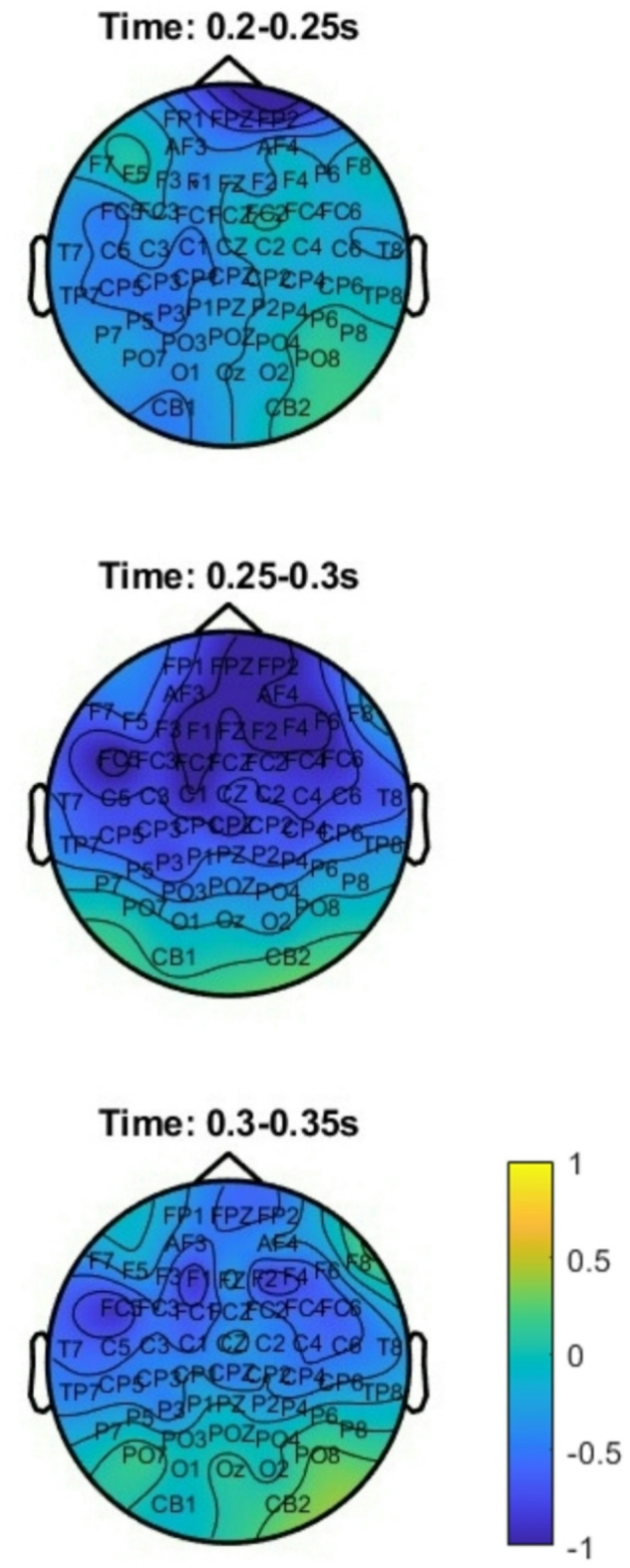
Topographic difference plots showing ERPs at the adverb for the 200 to 400 ms time window, for active-bias minus passive-bias condition; scale is from − 2 to 2 µV)

Figure 2 shows ERPs for all conditions whereas Fig. 3 shows ERPs at the adverb. Figure 4 shows topographic plots of the effects during the 200 ms to 400 ms time window at the adverb.

**Fig. 5:**
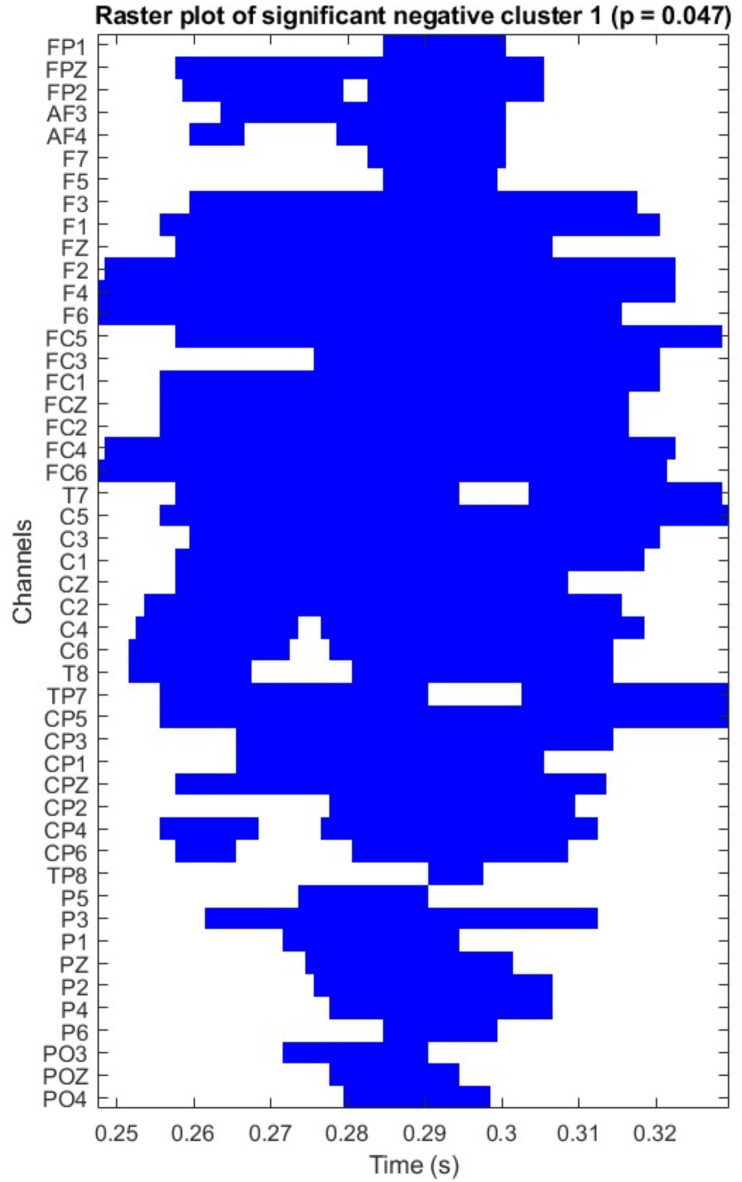
Raster plot showing which data points (i.e., which electrodes at which time points) were included in the cluster permutation-tested for the adverb. The raster plot in Fig. 5 can be read as follows: when a contrast between two conditions was significant, that significant difference was further identified as having been driven by differences at the channels and timepoints shown. For example, significant inter-condition differences in adverb ERP were driven mainly by differences between active-bias and passive-bias at around 250–330 ms and primarily at central and frontal channels.

The cluster-based permutation whole-epoch tests revealed a significant difference between the active-bias and passive-bias conditions. A broadly distributed negative shift (significant across 48 channels) was observed on the adverb (*p* = .043), with the cluster starting around 248 ms to 329 ms. Passive sentences with active-bias verbs elicited a larger negativity than those with passive-bias verbs did, particularly on midline electrodes and slightly left-lateralized (Fig. 3). From a latency and distribution perspective, the effect suggested an N280 component at the adverb (Figs. 2 and 3). As we summarized above, the N280 is an early negative-going component that peaks between 250-400ms post-stimulus with a predominantly left anterior distribution^42,68^, and it reflects grammatical processing complexity^42^. In our study, the ERP plots showed a peak occurring around 260-270ms, with a broadly distributed negativity that was particularly evident in frontal and central regions, which aligns with the typical N280 characteristics. To further confirm the presence of N280, we conducted an exploratory analysis, limiting the statistical test to the 250–400 ms time window, commonly used for the N280 component^42^. The cluster test was significant (*p* < .01) across the same 48 electrodes (Fig. 5).

The raster plot in Fig. 5 further highlights the specific channels and timepoints contributing to significant contrasts. Significant inter-condition differences in adverb ERPs were mainly driven by differences between active-bias and passive-bias at around 250–330 ms, and primarily at central and frontal channels.

## Discussion

The ERP results revealed that verb-bias significantly influenced sentence comprehension, with a negative shift observed at the post-verbal (adverb) region between 248 and 329 ms. This effect found in our study aligns with the N280, for grammatical processing complexity^42,71^ (unlike the LAN component found in previous studies^20,21^). It provides insights into the role of verb bias in thematic role processing in sentence comprehension. Our observed effects at the adverb, rather than the verb, suggest a spillover effect, where integration of verb bias and structural information continues beyond the critical word readers firstly encountered, which is commonly observed in ERP studies^72^. This finding supports incremental processing models, where integration of linguistic cues unfolds over time^73^.

The absence of effects at the NP2 or any effect in the positive polarity whole-epoch test aligns with the hypothesis that lexical biases influence the processing of passive sentences, and such a verb-structural mismatch appears to not require readers to engage in structural reanalysis. In SI, where passive structures are approximately as frequent and salient as active structures^74^, the processing of passive sentences with passive-bias verbs appears to be less cognitively demanding than those with active-bias verbs. However, it is important to acknowledge a limitation of the current study: we did not directly compare passive sentences to active sentences. Therefore, we cannot determine whether the observed effect represents a complete elimination of processing cost for passive sentences with passive-bias verbs, or merely a reduction in cost compared to active-bias verbs.

Additionally, we did not observe an N400 effect driven by the frequency difference between the verbs. This may partly reflect attenuated frequency-related effects in non-initial sentence positions^75,76^, a potentially medium-frequency nature^75^ of the verbs we used in this study, or potential impact of verb repetition across blocks^77^. Critically, our findings demonstrate that passive sentences with passive-bias verbs are processed much more efficiently than those with active-bias verbs, reflecting the influence of verb subcategorization preferences in shaping comprehension^6–9,11–13,17^.

## Concluding remarks

The current study investigated the effects of verb bias on language comprehension in Standard Indonesian, an Austronesian language with frequent and salient passive structures. We focused on the processing of passive sentences with active-bias and passive-bias verbs. This study sheds light on how verb bias influences passive sentence processing in Standard Indonesian, providing cross-linguistic evidence for the interplay between lexical subcategorization and syntactic complexity.

To our knowledge, it is the first ERP study of verb bias in passive structures, while manipulating verb bias. It expands existing research on sentence processing beyond active and object-first constructions. Second, it provides evidence that verb bias modulates thematic role assignment and syntactic integration in Indonesian, as reflected in early neural responses, like the N280. Third, it highlights the importance of language-specific features, such as structural frequencies, in investigating sentence processing difficulty.

Our findings also have broader theoretical implications. They support models of sentence comprehension emphasizing the interaction of lexical and syntactic information and highlight the role of structural frequencies in modulating processing costs. It also shows the N280 as a neural marker of syntactic complexity, extending its relevance to non-Indo-European languages. Future research could further examine how these effects generalize to other types of sentences’ processing, and other languages with varying structural frequencies.

## Electronic supplementary material

Below is the link to the electronic supplementary material.

Supplementary Material 1

## Data Availability

The dataset and codes used for this project will be publicly available via OSF at https://shorturl.at/G96VA. The full set of experimental items are shown in the appendix.
