# Supplementary material for: An ERP study on verb bias and thematic role assignment in standard Indonesian
Source: Sci Rep. 2025 Apr 7;15:11847. doi: 10.1038/s41598-025-96240-y (PMC11977004; doi:10.1038/s41598-025-96240-y)
Supplement: Supplementary file 1 — Supplementary Material 1 [file 41598_2025_96240_MOESM1_ESM.docx]

**Supporting Information**

**S1. Experimental Materials**

***Active-bias passive sentences***

Two versions of each experimental sentence were created with the NPs reversed, corresponding to the experimental conditions outlined in example (1) below.

1a. Pemuda itu ditampar kemarin oleh pria di jalanan

young.man the  PASS-slap  yesterday by man on  streets

“A young man was slapped yesterday by a man on the streets.”

1b. Pria itu ditampar kemarin oleh pemuda di jalanan

man the  PASS-slap  yesterday by young.man on  streets

“A man was slapped yesterday by a young man on the streets.”

Below items come with their approximate English translation. Please note that the materials in Indonesian do not contain number information (i.e. noun plurality), gender (policeman/woman or he/she), definiteness (i.e. articles), tense, and aspect. The articles shown in the English translation are not displayed in the original Indonesian materials.

| 2 | Pria itu ditendang kemarin oleh pemuda dari rumahnya  “The man was kicked yesterday by a youngster from his house” |
| --- | --- |
| 3 | Pejabat itu dihadang kemarin oleh gubernur di kantornya  “The official was confronted yesterday by a governor in his office” |
| 4 | Pria itu didorong kemarin oleh wanita saat masuk lift “The man was pushed yesterday by a woman while entering the elevator” |
| 5 | Pemuda itu diusir kemarin oleh wanita di kampusnya  “The youngster was ushered away yesterday by a woman on campus” |
| 6 | Staf itu didukung langsung oleh manajer karena pencapaiannya  “The staff was supported directly by the manager because of his/her achievements” |
| 7 | Perawat itu disambut langsung oleh dokter di rumah sakit  “The nurse was greeted directly by the doctor at the hospita” |
| 8 | Orang itu dipanggil langsung oleh staf agar masuk kantor  “The person was called directly by the staff to enter the office” |
| 9 | Pria itu diperintahkan kemarin oleh pemuda yang menjadi ketua  “The man was ordered yesterday by the youngster who was the chairman” |
| 10 | Pria itu dibimbing kemarin oleh pemuda dari rumahnya  “The man was tutored yesterday by a youngster from his house” |
| 11 | Pria itu dilatih kemarin oleh staf dari kantornya  “The man was trained yesterday by staff from his office” |
| 12 | Orang itu dikaji kemarin oleh peneliti di universitas  “The person was examined yesterday by a researcher at the university” |
| 13 | Wanita itu dikritik langsung oleh pria di kantor  “The woman was criticized directly by a man in the office” |
| 14 | Penjahat itu diseret kemarin oleh polisi karena perbuatannya  “The criminal was dragged yesterday by the police because of his actions” |
| 15 | Pria itu dibayar langsung oleh wanita untuk jasanya  “The man was paid directly by the woman for his services” |
| 16 | Wanita itu dikawal langsung oleh pria agar lebih aman  “The woman was directly escorted by a man for safety reasons” |
| 17 | Dokter itu ditikam kemarin oleh perampok pada siang hari  “The doctor was stabbed yesterday by a robber during the day” |
| 18 | Hakim itu diburu langsung oleh polisi karena kasus suap  “The judge was hunted directly by the police because of bribery” |
| 19 | Orang itu dibawa kemarin oleh petugas untuk jaga malam  “The person was taken yesterday by officers on night duty” |
| 20 | Pria itu diincar kemarin oleh wanita karena tindak criminal  “The man was targeted yesterday by the woman for a crime” |
| 21 | Wanita itu dijemput langsung oleh pria di mal  “The woman was picked up directly by the man in a mall” |
| 22 | Pria itu dihormati kemarin oleh pemuda karena tindakannya  “The man was respected yesterday by the youngster due to his actions” |
| 23 | Pemuda itu diajari kemarin oleh pria di kantor  “The youngster was taught yesterday by the man in the office” |
| 24 | Wanita itu diajak langsung oleh pria untuk ikut pesta  “The woman was invited directly by the man to join the party” |
| 25 | Wanita itu diantar kemarin oleh pria ke bandara  “The woman was taken yesterday by the man to the airport |
| 26 | Murid itu dipegang kemarin oleh ibu guru di sekolah  “The student was held yesterday by the teacher at school” |
| 27 | Pemuda itu dikritik kemarin oleh pegawai pada sore hari  “The young man was criticized yesterday by an employee in the afternoon” |
| 28 | Pria itu didukung kemarin oleh manajer di kantor  “The man was supported yesterday by the manager at the office” |
| 29 | Pria itu diusir kemarin oleh pemuda dari rumahnya  “The man was thrown out yesterday by a youngster from his house” |
| 30 | Pejabat itu diminta kemarin oleh gubernur untuk turut membantu |

“The official was asked yesterday by the governor to help”

***Passive-bias passive sentences***

Two versions of each experimental sentence were created with the NPs reversed, corresponding to the experimental conditions outlined in example (1) below.

1a. Pria  itu dipecat kemarin oleh wanita karena tindakannya

man the PASS-fire yesterday by  woman because his.actions

“A man was fired yesterday by a woman because of his actions.”

1b. Wanita itu  dipecat  kemarin  oleh pria karena  tindakannya

woman the PASS-fire yesterday by  man because her.actions

“A woman was fired yesterday by a man because of her actions.”

Below are other items with their approximate English translations. Please note that the materials in Indonesian do not contain number information (i.e. noun plurality), gender (policeman/woman or he/she), definiteness (i.e. articles), tense, and aspect. The articles shown in the English translation are not displayed in the original Indonesian materials.

| 2 | Pemuda itu diduga kemarin oleh pria karena telah mencuri  “The young man was suspected yesterday by a man of having stolen” |
| --- | --- |
| 3 | Pria itu digemari kemarin oleh wanita dari kantornya  “The man was fancied yesterday by the woman from the office" |
| 4 | Pria itu disela kemarin oleh wanita saat masuk lift  “The man was interrupted yesterday by the woman while entering the lift” |
| 5 | Pemuda itu diminati kemarin oleh wanita di kampusnya  “The youngster was desired yesterday by the women on campus” |
| 6 | Staf itu diberhentikan kemarin oleh pria karena perbuatannya  “The staff was dismissed yesterday by the man because of his actions” |
| 7 | Perawat itu disuntik langsung oleh dokter di rumah sakit  “The nurse was injected directly by the doctor at the hospital” |
| 8 | Orang itu disahkan langsung oleh staf sebagai pekerja tetap  “The person is endorsed directly by the staff as a permanent employee” |
| 9 | Pria itu dipajang kemarin oleh pemuda yang mendapatkan penghargaan  “The man was paraded yesterday by the youngster who got the award” |
| 10 | Pria itu diculik kemarin oleh pemuda dari rumahnya  “The man was kidnapped yesterday by a youngster from his home” |
| 11 | Pria itu dipulangkan kemarin oleh staf dari kantornya  “The man was sent home yesterday by staff from his office” |
| 12 | Wanita itu dijumpai kemarin oleh pria di kebun Binatang  “The woman was met yesterday by a man at the zoo” |
| 13 | Wanita itu diwawancara kemarin oleh pria di kantor  “The woman was interviewed yesterday by the man in the office” |
| 14 | Polisi itu diwajibkan langsung oleh tentara untuk membuat laporan  “The policeman was required directly by the soldier to write reports” |
| 15 | Pria itu dipecat langsung oleh wanita karena tindakannya  “The man was fired directly by the woman because of his actions” |
| 16 | Wanita itu dipinjami langsung oleh pria uang untuk berobat  “The woman was lent (money) directly by the man for medical treatment” |
| 17 | Dokter itu divonis kemarin oleh hakim pada siang hari  “The doctor was sentenced yesterday by a judge at noon” |
| 18 | Hakim itu dijerat langsung oleh pengacara karena kasus suap  “The judge was charged directly by a lawyer in a bribery case” |
| 19 | Pria itu disiagakan kemarin oleh pemuda untuk jaga malam  “The man was alerted yesterday by the youngster for the night watch” |
| 20 | Pria itu dinamakan kemarin oleh wanita sebagai pahlawan negara  “The man was declared yesterday by a woman as a national hero” |
| 21 | Wanita itu disebut langsung oleh pria dalam pidatonya  “The woman is mentioned directly by the man in his speech” |
| 22 | Polisi itu dihukum langsung oleh tentara akibat tindak criminal  “The policeman was punished directly by the soldier for criminal acts” |
| 23 | Pemuda itu diciduk kemarin oleh pria di jalanan  “The youngster was taken yesterday by the man on the street” |
| 24 | Wanita itu disebut langsung oleh pria dalam pidatonya  “The woman was mentioned directly by the man in his speech” |
| 25 | Wanita itu didampingi kemarin oleh pria di bandara  “The woman was accompanied yesterday by the man at the airport” |
| 26 | Pemuda itu dianggap kemarin oleh guru sebagai orang hebat  “The youngster was considered yesterday by the teacher as a great person” |
| 27 | Pemuda itu ditugaskan kemarin oleh pegawai pada sore hari  “The young man was assigned (tasks) yesterday by employees in the afternoon” |
| 28 | Penjahat itu ditodong kemarin oleh perampok dalam pertarungan  “The criminal was held at gunpoint yesterday by a robber in a fight” |
| 29 | Pria itu dipukuli kemarin oleh pemuda dari rumahnya  “The man was beaten yesterday by a youngster at his house” |
| 30 | Pejabat itu dilantik kemarin oleh gubernur di kantornya  “The officials were sworn in yesterday by the governor in his office” |

**S2. Supplementary Table: Verb Frequency List**

|  | **Active** | **Passive** | **AFC** | **PFC** | **Afreq** | **Pfreq** | **English** | **Freq Ratio** |
| --- | --- | --- | --- | --- | --- | --- | --- | --- |
|  | **Active-bias verbs** | | | | | | |  |
| 1 | menampar | ditampar | 13 | 15 | 3028 | 984 | slap | 3.08 |
| 2 | menendang | ditendang | 12 | 14 | 6538 | 2235 | kick | 2.93 |
| 3 | menghadang | dihadang | 12 | 13 | 8460 | 4313 | block | 1.96 |
| 4 | mendorong | didorong | 8 | 10 | 175963 | 30953 | push | 5.68 |
| 5 | mengusir | diusir | 11 | 12 | 19997 | 10745 | chase away | 1.86 |
| 6 | mendukung | didukung | 7 | 8 | 300715 | 103137 | support | 2.92 |
| 7 | menyambut | disambut | 9 | 10 | 87559 | 44591 | welcome | 1.96 |
| 8 | memanggil | dipanggil | 9 | 10 | 50610 | 45771 | call | 1.11 |
| 9 | memerintahkan | diperintahkan | 10 | 11 | 46220 | 20182 | instruct | 2.29 |
| 10 | membimbing | dibimbing | 11 | 12 | 17238 | 6584 | guide | 2.62 |
| 11 | melatih | dilatih | 10 | 11 | 40679 | 19621 | practice | 2.07 |
| 12 | mengkaji | dikaji | 10 | 11 | 29560 | 19242 | study | 1.54 |
| 13 | mengkritik | dikritik | 11 | 12 | 18173 | 7111 | criticize | 2.56 |
| 14 | menyeret | diseret | 12 | 13 | 12011 | 3784 | drag | 3.17 |
| 15 | membayar | dibayar | 8 | 10 | 132427 | 41936 | pay | 3.16 |
| 16 | mengawal | dikawal | 11 | 12 | 17207 | 10179 | escort | 1.69 |
| 17 | menikam | ditikam | 14 | 15 | 2079 | 1262 | stabbed | 1.65 |
| 18 | memburu | diburu | 11 | 12 | 16431 | 8138 | hunt | 2.02 |
| 19 | membawa | dibawa | 7 | 8 | 343743 | 118983 | bring | 2.89 |
| 20 | mengincar | diincar | 11 | 13 | 13453 | 4970 | aiming | 2.71 |
| 21 | menjemput | dijemput | 11 | 12 | 15215 | 7592 | pick up | 2.00 |
| 22 | menghormati | dihormati | 10 | 11 | 45042 | 14199 | honor | 3.17 |
| 23 | mengajari | diajari | 12 | 13 | 7843 | 4642 | teaching | 1.69 |
| 24 | mengajak | diajak | 8 | 9 | 124056 | 50332 | invite | 2.46 |
| 25 | mengantar | diantar | 11 | 12 | 21008 | 12041 | deliver | 1.74 |
| 26 | memegang | dipegang | 9 | 10 | 72701 | 25487 | hold | 2.85 |
| 27 | mengkritik | dikritik | 11 | 12 | 18173 | 7111 | criticize | 2.56 |
| 28 | mendukung | didukung | 7 | 8 | 300715 | 103137 | support | 2.92 |
| 29 | mengusir | diusir | 11 | 12 | 19997 | 10745 | chase away | 1.86 |
| 30 | meminta | diminta | 7 | 8 | 385164 | 107644 | request | 3.58 |
|  | **Mean** | | 10.1 | 11.3 | 78400 | 28255 | *Afreq/Pfreq* | 2.49 |
|  | **Passive-bias verbs** | | | | | | |  |
| 31 | memecat | dipecat | 13 | 11 | 5898 | 14548 | fire | 2.47 |
| 32 | menduga | diduga | 10 | 8 | 35767 | 136107 | guess | 3.81 |
| 33 | menggemari | digemari | 14 | 12 | 2748 | 10645 | like | 3.87 |
| 34 | menyela | disela | 15 | 14 | 1104 | 2362 | interrupt | 2.14 |
| 35 | meminati | diminati | 14 | 10 | 1542 | 29005 | interested | 18.81 |
| 36 | memberhentikan | diberhentikan | 13 | 12 | 5109 | 10026 | stop | 1.96 |
| 37 | menyuntik | disuntik | 15 | 14 | 1195 | 2429 | inject | 2.03 |
| 38 | mengesahkan | disahkan | 12 | 11 | 7083 | 24008 | validate | 3.39 |
| 39 | memajang | dipajang | 13 | 12 | 4382 | 6775 | display | 1.55 |
| 40 | menculik | diculik | 13 | 12 | 3186 | 6073 | kidnap | 1.91 |
| 41 | memulangkan | dipulangkan | 13 | 12 | 4440 | 8380 | repatriate | 1.89 |
| 42 | menjumpai | dijumpai | 12 | 10 | 8243 | 25800 | encounter | 3.13 |
| 43 | mewawancara | diwawancara | 16 | 14 | 425 | 2757 | interview | 6.49 |
| 44 | mewajibkan | diwajibkan | 11 | 10 | 16803 | 28013 | oblige | 1.67 |
| 45 | memecat | dipecat | 13 | 11 | 5898 | 14548 | fire | 2.47 |
| 46 | meminjami | dipinjami | 17 | 16 | 301 | 411 | borrow | 1.37 |
| 47 | memvonis | divonis | 13 | 11 | 4095 | 14157 | convict | 3.46 |
| 48 | menjerat | dijerat | 12 | 11 | 9830 | 12332 | ensnare | 1.25 |
| 49 | menyiagakan | disiagakan | 14 | 13 | 2475 | 3696 | alert | 1.49 |
| 50 | menamakan | dinamakan | 13 | 10 | 5329 | 31583 | name | 5.93 |
| 51 | menyebut | disebut | 8 | 7 | 124627 | 342595 | mention | 2.75 |
| 52 | menghukum | dihukum | 11 | 10 | 14971 | 25150 | punish | 1.68 |
| 53 | menciduk | diciduk | 15 | 14 | 993 | 1853 | scoop | 1.87 |
| 54 | menyebut | disebut | 8 | 7 | 124627 | 342595 | mention | 2.75 |
| 55 | mendampingi | didampingi | 8 | 7 | 118603 | 262570 | accompany | 2.21 |
| 56 | menganggap | dianggap | 10 | 9 | 32805 | 70215 | consider | 2.14 |
| 57 | menugaskan | ditugaskan | 13 | 11 | 5300 | 14580 | assign | 2.75 |
| 58 | menodong | ditodong | 16 | 15 | 697 | 1003 | pointing | 1.44 |
| 59 | memukuli | dipukuli | 14 | 13 | 2923 | 3919 | hit/beat | 1.34 |
| 60 | melantik | dilantik | 12 | 10 | 10184 | 29999 | appoint | 2.95 |
|  | **Mean** | | 12.7 | 11.2 | 18719.43 | 49271.13 | *Pfreq/Afreq* | 3.10 |

AFC: active verb frequency class

PFC: passive verb frequency class

Afreq: active verb token frequency

Pfreq: passive verb token frequency

English: approximate translation of the verbs
